# Depression and Social-Behavioral and Academic Functioning among Early Adolescents: The Role of Classroom Context

**DOI:** 10.1007/s10802-026-01435-0

**Published:** 2026-03-16

**Authors:** Xinyin Chen, Jiaxi Zhou, Dan Li, Junsheng Liu, Tong Zhou

**Affiliations:** 1 Human Development-Quantitative Methods Division, Graduate School of Education, University of Pennsylvania, 3700 Walnut St., Philadelphia, PA 19104-6216 USA; 2 School of Psychology, Shanghai Normal University, Shanghai, China; 3 School of Psychology and Cognitive Science, East China Normal University, Shanghai, China

**Keywords:** Depression, Classroom context, Social-behavioral and academic functioning, Adolescents’ development

## Abstract

Social context plays an important role in shaping the development of socioemotional functioning. This one-year longitudinal study examined the moderating effects of classroom peer acceptance of students with depression (CPASWD) on relations between individual depression and adjustment among early adolescents. Participants in this study were students (*N *= 2,153; 1,088 boys), initially in fifth grade (*M*age = 11 years), in elementary schools in China. Data on depression, peer preference, and social and school adjustment were obtained from multiple sources including self-reports, peer nominations, teacher ratings, and school records. Multilevel analysis revealed that depression was negatively associated with later social competence and academic achievement and positively associated with later behavioral problems in classrooms with lower CPASWD but the associations were nonsignificant or weaker in classrooms with higher CPASWD. The results suggest that CPASWD served as a protective factor that buffered against the development of further adjustment difficulties of adolescents with depressive symptoms. The study indicates the significance of classroom context for psychopathological development.

**Supplementary Information:**

The online version contains supplementary material available at 10.1007/s10802-026-01435-0.

## Introduction

Depression in childhood and adolescence is a significant phenomenon because it is not only stable over time but also contributes to difficulties in other adjustment areas, such as social relationships and academic performance (Chang & Kuhlman, 2022; Cicchetti & Toth, 1998; Johnson et al., 2018; McLeod et al., 2016). Children and adolescents with depression tend to display pessimistic thinking, passive, withdrawn, and helpless behaviors, and undesirable interpersonal styles, such as excessive talking about negative emotions and experiences, focusing on personal failures, and seeking negative feedback (e.g., Joiner & Timmons, 2009; Rose et al., 2007). As a result, they may experience difficulties in social interactions and in obtaining social support for performance on school tasks (Allen et al., 2014; Chen & Liu, 2016). Depressive symptoms, such as loss of concentration, persistent fatigue or loss of energy, and declined interest in daily life activities, may also impede cognitive functioning and interfere with the process of learning (Clayborne et al., 2019). The social-relational experiences and social-cognitive processes are likely to undermine the motivation to engage in social and academic activities in school and facilitate negative attitudes toward the school, making children and adolescents with depression vulnerable to difficulties in adjustment (Schwartz et al., 2005). Consistent with these arguments, it has been found that depressive symptoms are associated with low social status and peer victimization (e.g., Chen & Liu, 2016; Lin et al., 2008; Rubin et al., 2015). Depression in school-age children and adolescents is also predictive of low academic achievement and learning difficulties (Clayborne et al., 2019; McLeod et al., 2012; Lin et al., 2008).

According to the transactional view of development (Cicchetti, 2016; Masten & Cicchetti, 2010; Sameroff, 2009), the contributions of individual characteristics including depression occur in social context and thus are likely to be constrained by social-contextual factors. Consistent with this view, the contextual-developmental perspective (Chen, 2018, 2020) states that social context may play a role in shaping the functional meaning or adaptational significance of individual characteristics. For school-age children and adolescents, classroom represents an important social environment in which they interact with peers on a daily basis. During social interactions, students interpret and evaluate each other’s behaviors and express their attitudes and responses (e.g., approval or disapproval) toward those who display the behaviors. Social attitudes and responses related to specific behaviors constitute a classroom climate or norm that may have significant implications for individual adjustment and development. In the present longitudinal study with a

 sample of early adolescents in China, we sought to examine the role of classroom peer acceptance of students with depression (CPASWD) in the relations between individual depression and adjustment outcomes.

### Depression and Adjustment in Chinese Adolescents: A Background

In the traditional group-oriented Chinese society, the primary socialization goal is to help children develop behaviors and qualities that are conducive to harmonious interpersonal relationships and group functioning. In this context, children’s and adolescents’ psychological wellbeing has been considered unimportant and thus largely neglected (Chen, 2023). Moreover, displaying personal negative feelings in social interactions is regarded as inappropriate, selfish, and shameful because it may disrupt social harmony; individuals are encouraged to suppress their negative emotions and feelings in social settings (Liu et al., 2018; Russell & Yik, 1996). Consistent with the general cultural background, parents and teachers tend to be insensitive to depressive symptoms and other internalizing psychological problems of children as they are expected to concentrate on their school performance (e.g., Chen, 2010).

The massive social change in China over the past several decades has led to a rise of self-oriented values, which has pervasive implications for socialization and youth development. With urbanization and economic development, parental dependence on children for future financial support and other resources has decreased, which is associated with increased appreciation of children’s psychological needs and socioemotional wellbeing in the family and the society (Kagitcibasi, 2012). To help students develop abilities and qualities for better adaptation in the new highly competitive environment, schools in China have been required by the Ministry of Education to improve a balance of curricula to focus more on comprehensive development in academic as well as socioemotional areas, including those that have traditionally neglected (Cai et al., 2020). Interviews with Chinese parents and teachers showed that they believed children’s socioemotional functioning to be critical to their future success in the society and that they were concerned about children’s psychological health (e.g., Way et al., 2013; Zhou et al., 2018). Moreover, research has revealed that, compared with their Western counterparts, Chinese children and adolescents display equal, or even higher, levels of affect disturbances including depression (e.g., Zhong et al., 2013). Psychological problems have received heightened attention from professionals and the public in recent years as increased mental health incidents, such as suicide, in youth have been reported in the media (e.g., Caskie, 2018; Liu et al., 2020). Given this background, it is important to investigate adolescents’ depression and its adjustment outcomes in China from different perspectives.

### The Role of Classroom Context

The extent to which specific behaviors or attributes are perceived and evaluated in the group represents an important aspect of the social environment in which individuals interact with others (Chen, 2012; Laursen & Veenstra, 2021). Researchers have recently examined the role of classroom context or norms in shaping adolescent development (Garandeau et al., 2022; Hu et al., 2023; Horoz et al., 2024). Garandeau et al. (2022), for example, found that bullying defending behaviors were more popular in classrooms where bullying was considered more unacceptable.

In the present study, we were interested in how CPASWD served as a context in moderating the relations between individual depression and adjustment outcomes in adolescents. In the psychopathological literature, an interactional model that is commonly used for contextual effects is the stress-buffering model (Cohen & Wills, 1985; Masten & Wright, 1998; Rutter, 2013). This model focuses on factors that buffer against or exacerbate the effect of the risk or adversity. For example, in a classroom where depressive symptoms (e.g., passive and withdrawn behaviors, dwelling on negative emotions) are perceived as unacceptable and unfavorable, students with depression or students who display the symptoms are likely viewed as a misfit or deviant and may experience obstacles and difficulties in engaging in social interaction, establishing positive relationships, and obtaining social and emotional support from others. These students may be more vulnerable to adverse experiences such as social isolation and peer victimization. As a result, adolescents with depression in this environment may develop further psychological distress and other adjustment problems. In other words, being in a classroom with low CPASWD may increase the risk for adolescents with depression, exacerbating their adjustment problems.

On the other hand, in a classroom with higher CPASWD, depressive symptoms may be viewed as less problematic and deviant, and students with depression may not receive the same level of social disapproval or adverse treatment from peers. In this environment, peers may express sympathy and provide support to these students, which may help them cope with their emotional distress and enhance their confidence in social interaction. Thus, being in a classroom with high CPASWD may reduce the risk of adolescents with depression and thus protect them from developing further problems. Statistically, we would expect positive individual-level relations between depression and later depression and adjustment problems or negative individual-level relations between depression and later positive outcomes in classrooms with lower CPASWD and weaker or nonsignificant individual-level relations in classrooms with higher CPASWD.

### The Present Study

The main purpose of this one-year longitudinal study was to examine the role of CPASWD in moderating the relations between individual depression and later social-behavioral and school adjustment in Chinese adolescents. To our knowledge, this is the first study focusing on the role of classroom context related to depression. The study was conducted in a region consisting of rapidly developing towns, small cities, and surrounding areas. The mixed cultural values and lifestyles in the region (Zhao et al., 2024) might result in considerable variations in the views and attitudes toward depression, which provided an excellent opportunity to examine the effects of the group-level context on the associations between depression and adjustment at the individual level. A sample of students, initially in Grade 5, in elementary schools participated in the one-year longitudinal study. The years of Grades 5 and 6 are the final years in elementary school in China and represent a crucial period of development as students engage in increasingly extensive and intensive peer interactions and experience heighted pressure for social and academic achievement in school (e.g., Chen, 2023). At the same time, adolescents are becoming highly sensitive to social evaluations in school (Blakemore & Mills, 2014; Chen, 2012; Somerville, 2013).

As suggested by other researchers (e.g., Hu et al., 2023; Veenstra & Lodder, 2022), we measured the classroom depression-related context using the within classroom correlation between depression and peer preference. Previous studies have shown that the preference-based norm plays a significant role in adolescents’ development (Hei et al., 2025; Hu et al., 2023). According to Ladd and colleagues (e.g., Ladd et al., 2006), the adjustment of students is concerned with how they successfully adapt to the school environment, as indicated by their competence in addressing the various requirements of the school, such as performing on social and academic tasks. We collected data on students’ adjustment in domains of social competence, behavioral problems, peer victimization, and academic achievement. As our main hypotheses, we first expected that depression would be negatively associated with later social competence and academic achievement and positively associated with later behavioral problems and peer victimization. Furthermore, based on the previous discussion, we hypothesized that the associations would be more evident in classrooms with lower CPASWD.

## Method

### Participants

Participants in this study were fifth-grade students (*N* = 2,153; 1,088 boys) attending elementary schools in a region primarily consisting of towns, small cities, and surrounding areas in East China. This region is home to a population of approximately 2.7 million located in the lower Yangtze River drainage basin and Yangtze River Delta. The students were from 39 classes in five regular public schools. The average age of the participants was 11 years and 1 month (*SD* = 9.20 months). These public schools primarily served children from their respective local geographic regions. Stipulated by the Ministry of Education in China, the structure and organization of Chinese schools are similar. Students typically follow similar schedules of courses and activities, remain in the same class over the years, and are not permitted to switch classes. Each class is overseen by a head teacher who teaches a major course and manages the social and daily activities of the class and thus is very familiar with the students. Students are encouraged to engage in various social and academic activities in the school, which provides extensive opportunities for interaction among classmates.

Almost all the children (97%) were from two-parent families, and 51.8% of them were only children. The majority of participants came from families of low to middle socioeconomic backgrounds. In the sample, 82.84% of the fathers and 89.18% of the mothers had attained an education level of junior high school or below, 12.12% of the fathers and 7.02% of the mothers had a senior high school education, 4.09% of the fathers and 3.06% of the mothers had occupational or technical school training, and 0.94% of the fathers and 0.74% of the mothers had a college or above college education. Most participants were of the Han ethnic nationality, which constitutes over 90% of China’s population. The demographic variables had no significant effects on the variables or the relations in the study.

From the original sample, 1,962 children participated in a follow up study one year later (see Figure S1 in Supplementary Materials). Multivariate analysis of variance (MANOVA) revealed nonsignificant differences between the children who participated in the follow-up and those who did not, based on the variables at Time 1, Wilks’ λ = 0.996, *F*(6, 1480) = 1.07, *p* =.376.

### Measures

#### Depression

Students’ depression was assessed using a 14-item form of Chinese version of Kovacs’s *Childhood Depression Inventory* (1992). The items were concerned with a given thought, feeling or behavior associated with depression (e.g., self-blame, self-hate, fatigue, anhedonia, and reduced appetite). For each item, participants were asked to choose the one that best described them in the past 2 weeks from three alternative responses (e.g., “I feel like crying every day,” “I feel like crying most days,” “I feel like crying once in a while”). Response to each item was scored as 0, 1, and 2, with higher scores indicating greater depression. The average score of the items was computed, with higher scores indicating greater depression. The short form was used in this study because it was shown to be effective in assessing adolescents’ depressive symptoms and was reliable and valid in previous studies with Chinese students (e.g., Chen et al., 2024; Liu et al., 2023). The internal reliabilities (Cronbach’s α coefficients) of this measure were 0.80 and 0.81 at Times 1 and 2, respectively, in this study.

#### Peer Preference

Children were asked to nominate up to three classmates they most liked to be with, and three classmates they least liked to be with (positive and negative nominations). As suggested by other researchers (Coie et al., 1995), cross-gender nominations were allowed. For each student, the numbers of positive and negative nominations received from all classmates were summed separately and then each standardized by the classroom (z-scores) to adjust for differences in classroom size and the number of nominators. The standardized scores indicated the students’ relative standings on peer liking or disliking within the classroom and allowed for appropriate comparisons across classrooms. Following the procedure by Coie et al. (1995), an index of peer preference, indicating how well an individual is preferred by peers in the classroom, was formed by subtracting negative nomination scores from positive nomination scores. This sociometric measure was used and shown to be reliable and valid in Chinese students (e.g., Chen et al., 2005, 2024; Liu et al., 2023).

#### Classroom Peer Acceptance of Students with Depression (CPASWD)

Following the procedure used in previous research (Hu et al., 2023; Laninga-Wijnen et al., 2021), the CPASWD score was computed for each classroom using the Pearson correlation between students’ depression and peer preference scores. Higher values of CPASWD indicated that students with depressive symptoms were relatively more accepted or liked by their peers, reflecting the classroom context for the social experience of students with depression. Consistent with the previous research (e.g., Hu et al., 2023), the correlation was calculated based on all participants in the class; the measure represented the link between variations on depression and on peer preference among the students. This procedure has demonstrated good reliabilities and construct and predictive validities in previous studies (e.g., relations with social, school, and psychological adjustment; Hu et al., 2023; Laninga-Wijnen et al., 2021; Veenstra & Lodder, 2022).

#### Social Competence and Behavioral Problems

The head teacher of each class was asked to evaluate each student on social competence and behavioral problems, using a measure adapted from Hightower et al. (1986). Teachers were asked to rate each child on a 5-point scale, ranging from 1 (*not at all*) to 5 (*very well*), in terms of how well each item described the student. The measure comprised 20 items for social competence (e.g., “Participates in class discussion,” “Is friendly toward peers”) and 7 items for behavioral problems (e.g., “Disruptive in class,” “Overly aggressive to peers”). The item scores were standardized within the class to adjust for the teacher’s response style and to allow for appropriate comparisons. The average score was computed, with higher scores indicating higher levels of social competence or more behavioral problems. The measure was used and shown to be reliable and valid in previous studies with Chinese students (e.g., Chen et al., 2024; Zhao et al., 2024). The internal reliabilities of the measure in the present study were 0.90 and 0.92 for social competence and 0.78 and 0.77 for behavioral problems, at Times 1 and 2, respectively.

#### Peer Victimization

Peer victimization was assessed using a peer nomination measure (Schwartz et al., 2005). Participants were asked to nominate up to three classmates to fit each of four descriptors (e.g., “Gets picked on or teased by other kids,” “Is pushed or hit by other kids”). Nominations received from all classmates were summed to compute the item scores for each participant. The item scores were averaged and standardized within the class to form an index of peer victimization, with higher scores indicating higher peer victimization. The measure was used and shown to be reliable and valid in previous studies with Chinese students (e.g., Chen & Chen, 2020; Liu et al., 2017). The internal reliabilities of this measure were 0.78 and 0.80 at Times 1 and 2, respectively in the present study.

#### Academic Achievement

Data regarding academic achievement in Chinese, mathematics, and English—key subjects in Chinese schools—were obtained from school records. Each subject’s maximum score was 100, with 60 typically as the threshold for pass and fail. In the current study, the scores for Chinese, mathematics, and English were significantly correlated (*r* =.539 to 0.627, *p* <.001). Following procedures in previous studies (e.g., Fu et al., 2020), scores for these subjects were standardized within the class. The measure was used and shown to be reliable and valid in previous studies with Chinese children (e.g., Fu et al., 2020; Liu et al., 2017). The internal reliability coefficients for academic achievement in this study were 0.81 and 0.81 at Times 1 and 2, respectively.

### Procedure

We group administered the self-report measure of depression, the peer assessment measure of victimization, and a sociometric nomination measure to the students in the classroom during a regular 45-minute class period. Teachers were asked to complete a rating scale for each child assessing social competence and behavioral problems. Academic achievement data were obtained from school records. The study received approval from the Institutional Review Board at Shanghai Normal University. The participating schools were randomly selected through the local school board to represent typical public schools in the region. All fifth-grade classes in the schools were included and all students in the classes were invited to participate in the study with no criteria for exclusion. Active written assent was obtained from the participants and active written consent was obtained from their parents through the school. The participation rate was approximately 95% at each time. The measures were administered by a team of psychology faculty and graduate students in China. Detailed explanations of the procedures were provided during implementation. No evidence was found indicating that the students had difficulties understanding the procedures or the items in the measures.

### Data Analytic Plan

Multilevel models were conducted mainly to examine the moderating effect of CPASWD on the relations between Time 1 depression and each of the Time 2 adjustment variables (depression, social competence, behavioral problems, peer victimization, and academic achievement). In each model, Time 1 individual depression was included as an individual-level predictor, and child gender and the corresponding Time 1 adjustment variable (except in the model where depression was the outcome variable) were included as individual-level control variables. At the group level, Time 1 CPASWD was included as a predictor and Time 1 classroom size was included as a control variable. Cross-level interactions between Time 1 individual depression and CPASWD were included to examine how CPASWD moderated the associations between children’s Time 1 depression and the Time 2 adjustment variable. In the analyses, Time 1 individual depression and other Time 1 adjustment variables were group-mean centered.

When a significant interaction was identified, simple slope tests were conducted to examine the relations between Time 1 individual depression and Time 2 adjustment variable at high (1 SD above the mean) and low (1 SD below the mean) values of CPASWD, following the procedure suggested by Aiken and West (1991). The Johnson-Neyman technique was used to identify the significant regions where the simple slope of the regression line was significant.

## Results

### Descriptive Data

The Little’s MCAR test (Little, 1988) for the missing data (ranging from 0% to 30%) indicated that the data were missing completely at random, χ^2^(78) = 96.99 *p* =.071. As recommended by other researchers (Graham, 2009), the full information maximum likelihood (FIML) estimation was used to handle the missing data. A MANOVA revealed significant main effects of gender on the individual-level variables, Wilks’ λ = 0.79, *F*(11, 678) = 16.74, *p* <.001, 𝜂^2^ = 0.21. Subsequent univariate analyses indicated that, at both times, girls scored higher on social competence and academic achievement and lower on behavioral problems and peer victimization than boys. Additionally, girls had higher scores on peer preference at Time 1 and lower scores on depression at Time 1 compared to boys.

The means and standard deviations for boys and girls, as well as the correlations among the variables, are presented in Tables 1 and 2. The magnitudes of the correlations among the adjustment variables were weak to moderate, indicating that the measures tapped different but overlapping aspects of social, school, and psychological adjustment. The scores of classroom peer acceptance of students with depression (CPASWD) ranged from − 0.59 to 0.27 with the average score of −0.20 (*SD* = 0.19).

**Table 1:** Means and standard deviations of variables

	Boys		Girls		F-value
	Mean	SD	Mean	SD	
*Time 1*					
Depression	0.34	0.29	0.31	0.29	7.55**
Peer preference	−0.12	1.54	0.13	1.48	14.59***
Social competence	−0.20	0.95	0.20	0.99	85.86***
Behavioral problems	0.33	1.07	−0.34	0.77	267.40***
Peer victimization	0.13	1.10	−0.13	0.86	34.62***
Academic achievement	−0.19	0.91	0.19	0.77	77.44***
*Time 2*					
Depression	0.29	0.28	0.29	0.27	0.19
Social competence	−0.16	0.99	0.19	0.97	57.34***
Behavioral problems	0.34	1.08	−0.35	0.73	264.40***
Peer victimization	0.11	1.03	−0.13	0.89	30.20***
Academic achievement	−0.17	0.88	0.17	0.78	41.78***

** *p* <.01. *** *p* <.001

**Table 2:** Correlations among variables

	1.	2.	3.	4.	5.	6.	7.	8.	9.	10.
*Time 1*										
1. Depression										
2. Peer preference	− 0.20***									
3. Social competence	− 0.20***	0.25***								
4. Behavioral problems	0.14***	− 0.21***	− 0.12***							
5. Peer victimization	0.21***	− 0.55***	− 0.19***	0.28***						
6. Academic achievement	− 0.31***	0.34***	0.40***	− 0.23***	− 0.31***					
*Time 2*										
7. Depression	0.55***	− 0.14***	− 0.15***	0.05*	0.13***	− 0.22***				
8. Social competence	− 0.20***	0.21***	0.33***	− 0.14***	− 0.17***	0.39***	− 0.16***			
9. Behavioral problems	0.12***	− 0.21***	− 0.16***	0.37***	0.24***	− 0.18***	0.08***	− 0.22***		
10. Peer victimization	0.24***	− 0.49***	− 0.20***	0.23***	0.74***	− 0.31***	0.19***	− 0.20***	0.22***	
11. Academic achievement	− 0.23***	0.34***	0.40***	− 0.22***	− 0.28***	0.65***	− 0.20***	0.41***	− 0.25***	− 0.34***

* *p* <.05. ** *p* <.01. *** *p* <.001

### Multilevel Analyses

The results of the multilevel analyses, including the main effects of Time 1 individual- and group-level variables and the cross-level interaction between individual depression and CPASWD in predicting Time 2 adjustment variables, are presented in Table 3. The results showed that the stabilities of the adjustment variables from Time 1 to Time 2 were all significant. After controlling for individual Time 1 adjustment variable, classroom size, and gender, Time 1 depression was positively associated with Time 2 peer victimization and negatively associated with Time 2 social competence. The main effects of CPASWD on the Time 2 adjustment variables were nonsignificant.

**Table 3:** Effects of individual-level variables, group-level variables, and cross-level interactions on individual adjustment outcomes

T2 Outcome variable					
T1 Predictor		*B*	*SE*	*t value*	*95% CI*
Depression					
*Individual-level*					
Depression		0.482	0.028	16.931***	(0.426, 0.538)
*Classroom-level*					
CPASWD		−0.051	0.048	−1.064	(−0.146, 0.045)
*Cross-level interaction*					
Depression$\:\times\:$CPASWD		−0.221	0.102	−2.180*	(−0.420, −0.022)
Social competence					
*Individual-level*					
Social competence		0.284	0.023	12.082***	(0.238, 0.330)
Depression		−0.309	0.120	−2.588**	(−0.544, −0.075)
*Classroom-level*					
CPASWD		−0.050	0.119	−0.421	(−0.284, 0.183)
*Cross-level interaction*					
Depression$\:\times\:\:$CPASWD		0.929	0.422	2.201*	(0.101, 1.757)
Behavioral problems					
*Individual-level*					
Behavioral problems		0.271	0.023	11.934***	(0.227, 0.316)
Depression		0.023	0.113	0.206	(−0.198, 0.245)
*Classroom-level*					
CPASWD		−0.002	0.113	−0.017	(−0.224, 0.220)
*Cross-level interaction*					
Depression$\:\times\:$CPASWD		−0.932	0.400	−2.326*	(−1.717, −0.146)
Peer victimization					
*Individual-level*					
Peer victimization		0.671	0.016	42.238***	(0.640, 0.703)
Depression		0.258	0.078	3.286**	(0.104, 0.411)
*Classroom-level*					
CPASWD		0.003	0.003	1.063	(−0.003, 0.009)
*Cross-level interaction*					
Depression$\:\times\:$CPASWD		−0.280	0.279	−1.004	(−0.828, 0.267)
Academic achievement					
*Individual-level*					
Academic achievement		0.559	0.027	20.907***	(0.507, 0.612)
Depression		0.062	0.119	0.518	(−0.172, 0.295)
*Classroom-level*					
CPASWD		0.028	0.120	0.231	(−0.209, 0.265)
*Cross-level interaction*					
Depression$\:\times\:$CPASWD		1.049	0.430	2.440*	(0.205, 1.893)

*CPASWD = *classroom peer acceptance of students with depression. Gender and classroom size were controlled.

*p <.05. **p <.01. ***p <.001

**Fig. 1:**
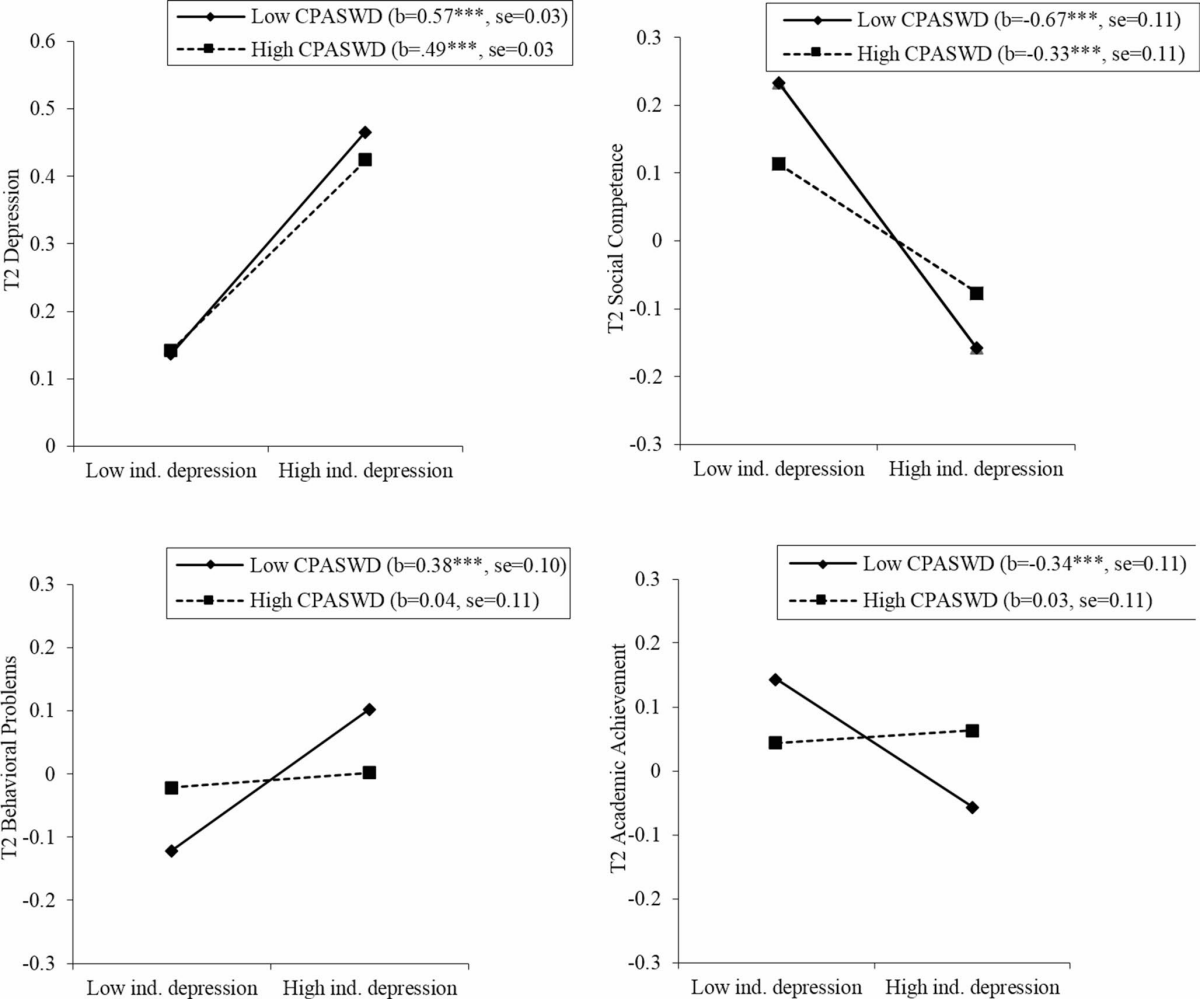
Simple slopes for interaction between Time 1 individual depression and CPASWD in predicting Time 2 adjustment variables. CPASWD = classroom peer acceptance of students with depression. *** *p* <.001

Significant interactions were found between Time 1 individual depression and CPASWD in predicting Time 2 depression, social competence, behavioral problems, academic achievement. Simple slope analyses following the procedure by Aiken and West (1991) indicated that Time 1 depression was significantly and positively associated with Time 2 depression in classrooms with lower CPASWD; this association was weaker in classrooms with higher CPASWD. Time 1 depression was significantly and negatively associated with Time 2 social competence in classrooms with lower CPASWD; the association was weaker in classrooms with higher CPASWD. Time 1 depression was significantly and positively associated with behavioral problems and negatively associated with academic achievement in classrooms with lower CPASWD, but these associations were not significant in classrooms with higher CPASWD. The results concerning the moderating effects are presented in Fig. 1.

**Fig. 2:**
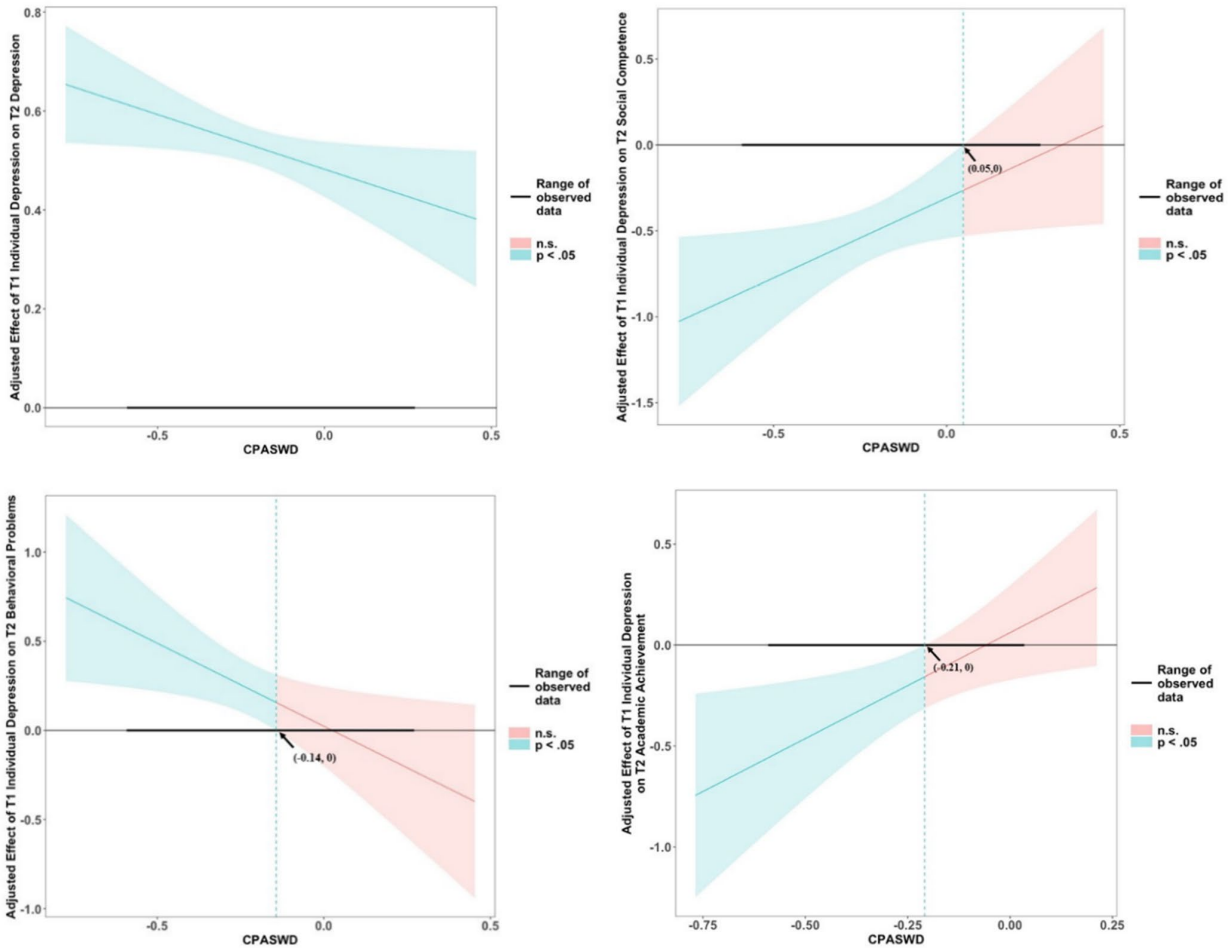
Adjusted effects of Time 1 individual depression on Time 2 adjustment variable as a function of CPASWD using the Johnson–Neyman technique. CPASWD = classroom peer acceptance of students with depression. The regions of significance are highlighted, indicating the bounds of the upper and lower 95% confidence intervals. Blue regions represent areas where the effect is statistically significant (*p* <.05), while red regions indicate that the effect is not statistically significant

The Johnson–Neyman technique was used to identify the significant regions for the interactions. As shown in Fig. 2, Time 1 depression was significantly positively associated with Time 2 depression across all classrooms in the sample. Time 1 depression was significantly and negatively associated with Time 2 social competence for children in classrooms where the CPASWD score was less than 0.05, suggesting that in classrooms with CPASWD scores lower than 0.05, students with depression at Time 1 were less socially competent at Time 2. Time 1 depression was significantly and positively associated with Time 2 behavioral problems for children in classrooms where the CPASWD score was less than − 0.14, suggesting that in classrooms with CPASWD scores lower than − 0.14, students with depression at Time 1 had more behavioral problems at Time 2. In addition, Time 1 depression was significantly and negatively associated with Time 2 academic achievement for children in classrooms where the CPASWD score was less than − 0.21, suggesting that in classrooms with CPASWD scores lower than − 0.21, students with depression at Time 1 were lower on academic achievement at Time 2. These results suggest that relatively high CPASWD served as a buffering factor, mitigating the development of negative social and school adjustment outcomes for adolescents experiencing depression.

We examined gender differences by including three-way interactions among Time 1 individual depression, CPASWD and gender in the models. The results indicated that the three-way interactions were nonsignificant, suggesting that the moderating effects of CPASWD on the relations between Time 1 individual depression and Time 2 adjustment variables were consistent for boys and girls.

## Discussion

It has been argued that social context plays an important role in shaping the developmental outcomes of socioemotional functioning (Cicchetti, 1998; Sameroff, 2009). Consistent with this argument, the results of the present study showed that classroom depression norm, as indicated by classroom peer acceptance of students with depression (CPASWD), had significant moderating effects on the development of individual depression and on its relations with adjustment. Specifically, whereas Time 1 depression predicted Time 2 depression, indicating the robust stability of depression over time, the stability was significantly weaker in classrooms with higher CPASWD. Moreover, Time 1 depression was negatively associated with Time 2 social competence and academic achievement and positively associated with Time 2 behavioral problems in classrooms with lower CPASWD but the association was non-significant or weaker in classrooms with higher CPASWD. The results suggest that in classrooms where students with depression had more positive experiences with classmates, these students were less likely to maintain depressive symptoms and to develop social and school problems. The favorable classroom depression-related context served as a protective factor that buffered against the development of further adjustment difficulties of adolescents with depression.

### The Moderating Role of CPASWD

The study provided evidence for the effect of CPASWD that is consistent with the stress-buffering model (Cohen & Wills, 1985; Rutter, 2013). The contributions of classroom context may involve social-relational and social-cognitive processes. In a context with low CPASWD, peers may be inclined to exhibit negative reactions during social interactions to adolescents displaying depressive symptoms, such as withdrawn and passive behaviors and expression of negative affect. As such, these adolescents are likely to experience heightened stress and distress, which may exacerbate their depressive symptoms and adjustment difficulties. The adverse experiences of adolescents with depression may elicit their negative attitudes toward the school and undermine their motivation and performance on schoolwork (e.g., Clayborne et al., 2019; Chang & Kuhlman, 2022; Johnson et al., 2018). On the other hand, in a context with high CPASWD, adolescents with depression may receive less negative treatment and greater social support in coping with their social and psychological difficulties. The supportive environment allows them to engage in more activities with others, which provide opportunities for them to learn social standards for appropriate behaviors and skills to control behavioral problems and acquire and display social competence (e.g., Rubin et al., 2015). Social support and instrumental assistance that they receive may also help them improve their learning capacity and performance on academic tasks. In short, the classroom context with high CPASWD may reduce the risk of adolescents with depression and protect them from developing further problems over time.

From the social salience perspective (Hartup, 1996; Laninga-Wijnen et al., 2021; Veenstra & Lodder, 2022), a behavior or attribute is likely to be significant in predicting individual adjustment outcomes when it associated with social approval or disapproval in the peer group. CPASWD may be viewed as related to the social salience of depression in the classroom context. In classrooms with lower CPASWD, depressive behaviors may be more socially salient with negative valence in eliciting undesirable peer evaluations and responses, making depression a more evident risk factor in the development of difficulties in social, school, and psychological domains. The classroom context with higher CPASWD may serve to attenuate the salient adverse effects of depression. The moderating effects of CPASWD may involve other processes. For example, a context in which students with higher depression are relatively liked may be related to normalization of depressive symptoms, in which peers approve and perhaps reinforce the symptoms, such as displaying pessimistic thinking styles and helpless behaviors. The problematic normalization may also be related to corumination, focusing on excessive talking about negative emotions and experiences (Rose et al., 2007), which tends to exacerbate the difficulties of adolescents with depression. Although this argument does not appear consistent with the results that adolescents with depression developed less social and psychological problems in classrooms with higher CPASWD in the present study, corumination, contagion, and related issues should be explored in future research.

The results of the study have important practical implications. Teachers and professionals should be aware of the classroom environment and the potential effects of CPASWD on students with depressive symptoms. In classrooms where depression is highly unfavored or disliked, teachers should pay particular attention to the difficulties that students with depression may experience and develop strategies to help them improve their social and academic performance. It may be an effective strategy to design classroom norm-based educational and intervention programs with a focus on creating an inclusive setting to enhance social understanding and support for students with depression to engage in social activities and interaction with peers.

### Limitations and Future Directions

Several limitations and weaknesses in the study should be noted. First, although we discussed the processes involved in the contributions of classroom context, such as social evaluations, based on the literature (e.g., Chen, 2012; Somerville, 2013), these processes were not examined in the study. Social evaluations are concerned with individual judgments of and responses to the display of depressive symptoms, which reflect peers’ attitudes toward students with depression and constitute a basis for the formation of CPASWD. Researchers should explore the processes in future research. Behavioral observations may provide valuable information about how students with depressive symptoms interact with peers in different classrooms. Interviews with peers about their attitudes toward depression may also help understand the social–cognitive processes involved their interaction with students with depressive symptoms. Relatedly, adolescents’ depression may manifest in different behavioral forms, such as social withdrawal, passivity, seeking support, and disruptive behaviors, which may bring about different evaluations and reactions from peers. It will be important to investigate specific behavioral expressions of depression and their relations with peer evaluations and individual adjustment outcomes. It will also be important to examine how other contextual factors, such as classroom- or school-level characteristics (e.g., teacher characteristics and school priorities) interact with peer attitudes in their contributions to adolescents’ depression.

Second, the present study focused on the role of classroom context regarding depression in individual development. It will be interesting to investigate how social and individual factors contribute to the development of classroom context.

Third, in this study, students’ social competence and behavioral problems were both rated by the head teachers, which may introduce shared method variance or rater biases. This concern might be partly mitigated by the head teachers’ extensive daily interactions with the students in the class, which allowed the teachers to evaluate students’ competence and behavioral problems relatively accurately. Nevertheless, one should be careful in interpreting the results concerning students’ social competence and behavioral problems in this study.

Fourth, the present study was conducted in a region consisting mostly of towns, small cities, and surrounding areas in East China. Substantial differences exist across regions in China in social and economic development. Thus, generalization of the results to other regions, such as large cities and more remote rural areas, needs to be made with caution. In addition, the study was conducted in a sample of early adolescents. One needs to be careful in generalizing the results to other developmental periods, such as middle childhood and middle and late adolescence.

Fifth, two waves of longitudinal data over one year were collected in this study. Researchers should collect multi-wave data over a longer period of time in the future to provide a more comprehensive understanding about the developmental patterns of depression in social contexts.

Finally, whereas the findings of the present study generally mesh well with the literature on the role of social-ecological settings in the development of psychopathological functioning (e.g., Bronfenbrenner & Morris, 2006; Cicchetti & Toth, 1998), the Chinese context may need to be considered in understanding specific results. For example, group-orientation and social connectedness that are emphasized in Chinese society (Chen, 2023) may strengthen the effects of classroom norms. It will be important to investigate whether and how classroom context plays a role in shaping the development of adolescent depression in societies such as the United States where independence and self-orientation are encouraged.

## Supplementary Information

Below is the link to the electronic supplementary material.

Supplementary Material 1 (DOCX 24.5 KB)

## Data Availability

The data and material for the current study are not publicly available but are available from the corresponding author on reasonable request.
