# Supplementary material for: Depression and Social-Behavioral and Academic Functioning among Early Adolescents: The Role of Classroom Context
Source: Res Child Adolesc Psychopathol. 2026 Mar 16;54(2):48. doi: 10.1007/s10802-026-01435-0 (PMC12992467; doi:10.1007/s10802-026-01435-0)
Supplement: Supplementary file 1 — Supplementary Material 1 (DOCX 24.5 KB) [file 10802_2026_1435_MOESM1_ESM.docx]

**Figure S1. Flow Diagram for Study Participants**

Participated in the initial study (Time 1)

*N* = 2,153

Participated in follow-up study (Time 2)

*N* = 1,962

Did not participate in follow-up study (Time 2)

*N* = 191

Included in final analyses using full information maximum likelihood (FIML) estimation

*N* = 2,153
